# Prevalence and Predictors of Insufficient Plasma Vitamin C in a Subtropical Region and Its Associations with Risk Factors of Cardiovascular Diseases: A Retrospective Cross-Sectional Study

**DOI:** 10.3390/nu14051108

**Published:** 2022-03-06

**Authors:** Yao-Tsung Lin, Li-Kai Wang, Kuo-Chuan Hung, Chia-Yu Chang, Li-Ching Wu, Chung-Han Ho, Jen-Yin Chen

**Affiliations:** 1 Department of Anesthesiology, Chi Mei Medical Center, Tainan 71004, Taiwan; anekevin@hotmail.com (Y.-T.L.); anesth@gmail.com (L.-K.W.); ed102605@gmail.com (K.-C.H.); 2 Department of Hospital and Health Care Administration, Chia Nan University of Pharmacy and Science, Tainan 71710, Taiwan; 3 Department of Neurology, Chi Mei Medical Center, Tainan 71004, Taiwan; chiayu.chang7@msa.hinet.net; 4 The Center for General Education, Southern Taiwan University of Science and Technology, Tainan 80424, Taiwan; 5 Center for Precision Medicine, Chi Mei Medical Center, Tainan 71004, Taiwan; 540012@mail.chimei.org.tw; 6 Institute of Biomedical Sciences, National Sun Yat-sen University, Kaohsiung 80424, Taiwan; 7 Department of Medical Research, Chi Mei Medical Center, Tainan 71004, Taiwan; ho.c.hank@gmail.com

**Keywords:** insufficient plasma vitamin C, prevalence, cardiovascular diseases, risk factors, dyslipidemia, lipid-independent markers, homocysteine, C-reactive protein, lipoprotein(a)

## Abstract

Background: to evaluate the prevalence and predictors of insufficient plasma vitamin C among adults in a subtropical region and its associations with cardiovascular disease risk factors including dyslipidemia and lipid-independent markers, namely homocysteine, high-sensitivity C-reactive protein (hs-CRP) and lipoprotein(a). Methods: Data of this retrospective cross-sectional study were extracted from electronic medical database of a Medical Center. Based on plasma vitamin C status, subjects were split into two groups—subjects with sufficient and insufficient plasma vitamin C levels (<50 µmol/L, ≤8.8 mg/L). Results: Prevalence of insufficient plasma vitamin C in 3899 adults was 39%. Multivariate logistic regression identified male gender, high body mass index, age 20–39, and winter/spring as independent predictors of insufficient vitamin C among all subjects. Greater proportions of subjects with insufficient plasma vitamin C had lower high-density lipoprotein cholesterol levels and elevated levels of triglyceride, homocysteine and hs-CRP (all *p* < 0.001). There were no differences in total cholesterol, low-density lipoprotein cholesterol and lipoprotein(a) between groups. Conclusions: There was a high prevalence of insufficient plasma vitamin C in the subtropical region, which indicates that insufficient plasma vitamin C remains a public health issue. Further study is needed to confirm these findings and to determine the underlying mechanisms.

## 1. Introduction

Cardiovascular diseases are the leading cause of death worldwide. However, cardiovascular disease is largely preventable. It is critical to prevent cardiovascular diseases by addressing risk factors which can be modifiable and nonmodifiable. Dyslipidemia, including high triglycerides, elevated levels of low-density lipoprotein cholesterol (LDL-C) and low levels of high-density lipoprotein cholesterol (HDL-C), is an established risk factor for cardiovascular diseases [1]. Hyperhomocysteinemia [2], elevated lipoprotein(a) [3] and high C-reactive protein (CRP) [4] are lipid-independent risk factors for cardiovascular diseases. Interestingly, all of these risk factors for cardiovascular diseases are associated with oxidative stress and may be modified.

Vitamin C, also known as ascorbic acid, is a powerful antioxidant and an essential micronutrient which is required for many physiological and biological functions. However, humans are unable to synthesize vitamin C endogenously due to lack of enzyme gulonolactone oxidase as a result of a mutation occurring approximately 40 million years ago [5]. Vitamin C deficiency leads to hypertriglyceridemia and vitamin C supplementation significantly lowers plasma triglyceride levels in guinea pigs [6,7]. LDL-C oxidation increases its atherogenesis in the intima of blood vessels [8]. HDL-C has important cardioprotective effects by the protection of LDL-C from oxidative modification and the reverse cholesterol transport [9]. Although HDL-C has antiatherogenic properties, HDL-C is susceptible to oxidation, which reduces its ability to protecting LDL-C from oxidative modification [10]. Importantly, vitamin C can protect HDL-C and LDL-C from oxidation [11,12,13]. Hence, vitamin C deficiency may increase the risk of dyslipidemia and atherosclerotic development. Among the vast biological functions of vitamin C, it is suggested to play a role in primary prevention of cardiovascular diseases.

Hyperhomocysteinemia has been identified as a strong lipid-independent risk factor for atherosclerotic vascular disease [14]. Homocysteine not only reduces the HDL-C production but also promotes LDL-C oxidation [12,15,16]. As a strong antioxidant, plasma vitamin C levels was found to be negatively associated with homocysteine levels [17]. Elevated lipoprotein(a) is also a lipid-independent risk factor for cardiovascular diseases through stimulation of platelet aggregation and promotion of endothelial dysfunction and phospholipid oxidation [3]. In a transgenic mice model, vitamin C deficiency was found to increase serum lipoprotein(a) levels and promote vascular accumulations of lipoprotein(a) resulting in atherosclerotic changes [18]. CRP, a biomarker of inflammation, is a mediator of atherosclerosis [4]. Supplementation of vitamin C decreases elevated CRP [19]. Taken together, plasma vitamin C levels may have implications for these risk factors of cardiovascular diseases through the interaction and modification. Thus, a whole survey on the correlations between plasma vitamin C and the lipid-related and lipid-independent markers was needed.

A recent review shows that insufficient vitamin C remains common and an important factor in the global burden of diseases [20]. The geographic factor is one of key factors for vitamin C levels. Reports on the prevalence and predictors of insufficient plasma vitamin C of healthy subjects in subtropical regions are limited. The primary aim of the current study was to assess the prevalence and predictors of insufficient plasma vitamin C levels on adults aged 20 and older in a subtropical region. Secondary outcomes were to examine how plasma vitamin C status of the study population may be related to the aforementioned lipid-related and lipid-independent risk factors of cardiovascular diseases.

## 2. Materials and Methods

This retrospective cross-sectional study was approved by the Institutional Review Board of the Chi Mei Medical Center, a 1200-bed tertiary referral hospital in Tainan, Taiwan, which is located in a subtropical region and is known as ‘‘a fruit kingdom’’ [21]. The study was conducted in accordance with the Declaration of Helsinki.

### 2.1. Study Population

The periodic health examination in adults is an insured service of Taiwan national health insurance which provides universal, mandatory coverage with a rate up to 99% of Taiwan’s entire population by the end of 2004 [22]. Chi Mei Medical Center provides integrative medical healthcare options [23,24] involving nutrition surveys on serum/plasma vitamins A, B, C, D, E and trace minerals which are not insured. Data of subjects undergoing health examinations at our hospital were routinely stored in the electronic database of the institute. Health examination data at Chi Mei Medical Center between 1 January 2018 and 31 December 2019 were retrospectively collected and analyzed. Inclusion criteria for the current study were: (1) adult subjects aged 20 and older; (2) those with available data of plasma vitamin C, serum homocysteine, high-sensitivity CRP (hs-CRP), lipoprotein(a), triglyceride, total cholesterol, LDL-C and HDL-C examinations during the study period.

Exclusion criteria were: (1) subjects with diabetes or prediabetes (International Classification of Diseases, ICD) (ICD-9 250; ICD-10 E10-E14; R73.09 or HbA1C ≥ 5.7% [25,26]) which are confounders in the relationship of dyslipidemia and vitamin C status [27]; (2) individuals who had diagnostic codes of human immunodeficiency virus infection (HIV) [28] (ICD-9 042, 043, 044; ICD-10 B20-B24), solid organ transplantation [29] (ICD-9 3751, 1160, 1164, 1169, 5059, 5280, 5283, 5569, 3350–3352; ICD-10 Z94), chronic liver diseases (ICD-9 571; ICD-10 K70-77) [30] and/or chronic renal failure (ICD-9 585.5, 585.6; ICD-10 N18.5, N18.6) [31], which are potential confounding factors in the relationship of dyslipidemia and vitamin C status; (3) adults whose medical records showed no evidence of plasma vitamin C and other targeted examinations during the study period. Taiwan began using ICD-10-CM as the replacement for ICD-9-CM in 2016. In our hospital, an autosearch for the target diseases can be conducted by ICD-10 codes or ICD-9 codes which are automatically converted to ICD-10 codes.

Before health examinations, all subjects were required to fill in self-reported questionnaires which were used to collect data including job types, personal habits (smoking, drinking, and sleeping hours), and other items. However, completing the survey questionnaires was voluntary.

### 2.2. Study Parameters, Definitions and Cutoffs

In current international and national recommendations, a fasting plasma vitamin C concentration below 50 µmol/L (8.8 mg/L) is an indicator of insufficient levels [32]. Five categories of plasma vitamin C were defined: deficiency (≤2.0 mg/L, 11.4 μmol/L); subdeficiency (2.1–6.0 mg/L, 11.4–34.2 μmol/L); suboptimal (6.1–8.8 mg/L, 34.3–49 µmol/L); optimal (8.9–15 mg/L, 50–85 µmol/L); and supraphysiological (>15 mg/L, 85 µmol/L) [33,34,35]. Insufficient levels for vitamin C include deficiency, subdeficiency and suboptimal levels of vitamin C. Sufficient levels for vitamin C include optimal and supraphysiological levels.

The associations between plasma vitamin C concentrations and gender/age/season/body mass index (BMI) in the study population were investigated.

To evaluate the influence of age on plasma vitamin C concentrations, all eligible subjects were divided into three age groups: 20–39, 40–59 and ≥60 [35]. In Taiwan, seasonal fruits in winter are oranges and apples, with 41.2 mg and 2.9 mg of vitamin C per 100 g, respectively. Seasonal fruits in spring are pineapples and bananas, with 12.0 mg and 10.7 mg of vitamin C per 100 g, respectively. The summer fruits are lychees, mangos, pears and grapes with 52.3 mg, 22.7 mg, 4.6 mg and 3.8 mg vitamin C per 100 g, respectively. The autumn fruits are guavas, papayas, pomelos and pitayas with 137.9 mg, 58.3 mg, 54.5 mg and 5.3–6.3 mg vitamin C per 100 g, respectively (https://consumer.fda.gov.tw). The problem of insufficient plasma vitamin C concentration may be diet-based. According to vitamin C amount per 100 g in seasonal fruits, the seasons were categorized into two groups—higher vitamin C amounts in seasonal fruits (i.e., summer and autumn) and lower vitamin C amounts in seasonal fruits (i.e., winter and spring) to examine the association between seasons and plasma vitamin C concentrations. BMI was calculated as the weight in kilograms divided by the square of height in meters (kg/m^2^). Based on the WHO Asian BMI categories, all subjects in the present study were divided into those who were obese (i.e., BMI ≥ 27.5), overweight (23 ≤ BMI ≤ 27.4) and normal (BMI < 23) [36] to assess the relationship of BMI and plasma vitamin C status.

The ideal triglycerides, total cholesterol and LDL-C levels for an adult are 150 mg/dL, 200 mg/dL and 100 mg/ dL or less, respectively. The optimal cutoff value for HDL-C in predicting the risk of cardiovascular diseases is considered to be 46 mg/dL in the Taiwanese population [37]. The recommended homocysteine level is less than 10 µmol/L [38]. The lipoprotein(a) level (≥30 mg/dL) and hs-CRP (> or =1.0 mg/L) indicate elevated cardiovascular risk [19,39].

### 2.3. Assessment of Plasma Vitamin C, Serum Homocysteine, hs-CRP, Lipoprotein(a) and Lipid Profiles

#### 2.3.1. Blood Collection and Determination of Plasma Vitamin C Concentrations

A fasting blood sample of each subject was drawn in the morning during the day of the participants’ stay in hospital. The plasma sample after centrifugation was stored at a temperature of −80 °C, because the examination of the plasma vitamin C levels was done not all at once but after the date of hospital stay. Using a chromatograph Acquity UPLC Waters^®^ [40], plasma vitamin C quantification was usually performed once a week.

#### 2.3.2. Blood Collection and Determination of Serum Homocysteine

A fasting blood sample of each subject was drawn and stored at 2–8 °C after centrifugation. Serum levels of total L-homocysteine was determined by using chemiluminescent microparticle immunoassay technology on the ABBOTT Architect i2000SR System [41]. The measurement of homocysteine was completed within 4 h after blood sampling.

#### 2.3.3. Blood Collection and Determination of Serum hs-CRP, Lipoprotein(a) and Lipid Profiles

After an overnight fast, the blood samples of the subjects to measure the levels of hs-CRP, lipoprotein(a), triglyceride, total cholesterol, LDL-C and HDL-C were collected in the morning. The measurement of hs-CRP, lipoprotein(a), triglyceride, total cholesterol, LDL-C and HDL-C was completed within 4 h after the blood collection. Serum levels of hs-CRP were measured by immunoturbidimetric method (Abbott, Architect C-16000 analyzer) [42]. Our laboratory use Denka reagent kits and Denka kits to detect lipoprotein(a) in serum by Turbidimetric Immunology technology on the ABBOTT ARCHITECT c16000 analyzer. [43]. Serum levels of triglycerides, total cholesterol, HDL-C and LDL-C were determined enzymatically using an Abbott Architect c16000 clinical autoanalyzer.

### 2.4. Statistical Analysis

Data processing and statistical analysis were performed using SAS statistical software (Version 9.4; SAS Institute, Cary, NC, USA). Based on plasma vitamin C status, all subjects were grouped into two groups—subjects with sufficient and insufficient plasma vitamin C levels (<50 µmol/L, ≤8.8 mg/L). The difference of continuous data between groups was conducted by Student *t* test. Chi-square test was used to determine the significance of differences in categorical variables among groups.

Univariate logistic regression analysis was used to identify predictors of insufficient vitamin C. Confounding factors including gender, age groups, and seasons were adjusted to evaluate the odds ratio in different models. Identified variables that were associated with insufficient vitamin C (*p* < 0.10) in univariate analyses were entered into a multivariate logistic regression analysis. Independent predictors of insufficient vitamin C were presented as adjusted odds ratios (OR) and 95% confidence intervals (CI). A two-sided *p* value < 0.05 was significant.

## 3. Results

### 3.1. Demographic and Anthropometric Characteristics of the Study Population

After application of the inclusion and exclusion criteria, a total of 3899 subjects were included in the analysis. Demographic and anthropometric characteristics of the study population according to plasma vitamin C status are shown in Table 1.

The results demonstrated 39% of all subjects had insufficient plasma vitamin C levels (<50 µmol/L, ≤8.8 mg/L). Participants with insufficient plasma vitamin C levels were younger (47.6 years (SD 11.8 years), *p* < 0.001) and had higher BMI (24.90 (SD 3.86), *p* < 0.001). The prevalence of insufficient plasma vitamin C was higher in males (68.7%, *p* < 0.001). The proportions of subjects with insufficient plasma vitamin C were significantly different among three age groups (*p* = 0.001). The percentages of subjects with insufficient plasma vitamin C in four seasons were listed from high to low as spring (43.8%), winter (41.3%), summer (37.3%) and autumn (34.7%), which seemed to be associated with vitamin C amounts per 100 mg in seasonal fruits. As for adiposity, adults with greater BMI had a decreased tendency in plasma vitamin C status (*p* < 0.001).

### 3.2. Primary Outcomes

#### 3.2.1. Prevalence of Insufficient Plasma Vitamin C among Adults

**Figure 1:**
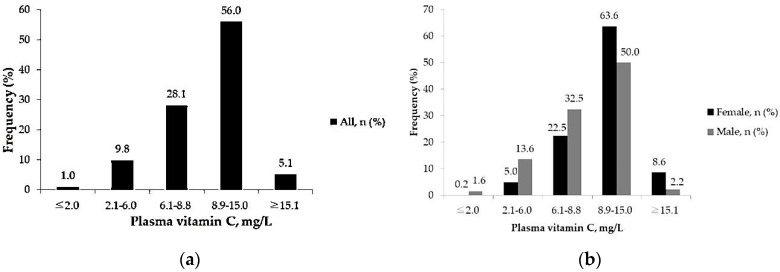
(**a**) Distribution of plasma vitamin C concentrations among all adults in a subtropical region; (**b**) Distribution of plasma vitamin C concentrations in female and male adults in a subtropical region.

According to five categories of plasma vitamin C, more than half of the participants had sufficient plasma vitamin C levels (≥50 µmol/L, ≥8.9 mg/L), composed of 56.0% with optimal and 5.1% with supraphysiological plasma vitamin C levels (i.e., vitamin C ≥ 15.1 mg/L). Insufficient levels of plasma vitamin C (<50 µmol/L, ≤8.8 mg/L) was found in 39% (1520) of the subjects, with deficient (≤2.0 mg/L), subdeficient (2.1–6.0 mg/L) and suboptimal (6.1–8.8 ng/mL) being noted in 1.0% (40), 9.8% (383) and 28.1% (1097) of the study population, respectively (Figure 1a).

Subjects with sufficient plasma vitamin C (≥8.9 mg/L) was more common in females than in males (72.2% vs. 52.2%, respectively, *p* < 0.001). This indicates that males had a dominant tendency of having insufficient plasma vitamin C (47.8% in males and 27.8% in females, *p* < 0.001) (Figure 1b).

#### 3.2.2. Predictors for Insufficient Vitamin C Levels among All Subjects

The correlations between the risks of insufficient plasma vitamin C status and demographic/environmental/anthropometric factors (i.e., gender, age, season, and BMI) in 3899 adults are presented in Table 2. Univariate logistic regression revealed a male predominance of insufficient vitamin C (crude OR: 2.37, 95% CI: 2.07–2.71). There was a trend of increasing prevalence of insufficient vitamin C with decreasing ages with the prevalence lowest (35.5%) in the elderly age group (≥60) and highest (44.1%) in the youngest age group (20–39). With reference to that of the ≥60 age group, only the crude OR of insufficient vitamin C for the 20–39 age groups was significantly higher (1.44, 95% CI: 1.18–1.75). There was also a significant seasonal variation in insufficient vitamin C levels with an increased risk in winter/spring compared to that in summer/autumn (crude OR 1.31, 95% CI: 1.16–1.50). In addition, a significant association was also found between BMI and insufficient vitamin C that higher BMI with higher insufficient vitamin C prevalence. The crude OR were 2.78 (95% CI: 2.30–3.35) in the obesity group (BMI ≥ 27.5) and 1.61 (95% CI: 1.39–1.86) in the overweight group (BMI: 23–27.4), respectively. According to the adjusted OR in multivariate logistic regression analysis, the risk factors for insufficient vitamin C were listed from high to low as male gender, high BMI (obesity and overweight), age 20–39, and winter/spring as significant predictors of insufficient vitamin C for all subjects in subtropical Taiwan.

#### 3.2.3. Predictors of Insufficient Vitamin C Levels among Female and Male Subjects

Subgroup analysis by genders, the findings in the female group were similar to that in all subjects (Table 2). Multivariate logistic regression analysis identified the significant predictors for insufficient vitamin C as high BMI (adjusted OR 2.96 for obesity, 1.42 for overweight), 20–39 age group (adjusted OR 2.21) and winter/spring (Adjusted OR 1.47) (Table 3a).

The findings in the male group were slightly different compared to the female group. Multivariate logistic regression revealed that obesity (BMI ≥ 27.5) was statistically significant with adjusted OR: 1.85 (95% CI: 1.44–2.37, *p* < 0.001), but overweight (BMI 23–27.4) was not with adjusted OR: 1.21 (95% CI: 0.98–1.48, *p* = 0.073). Winter/spring and the young age group (20–39) were still the risk factors (adjusted OR 1.33 (95% CI: 1.12–1.57); adjusted OR 1.31, 95% CI: (1.01–1.71), respectively) (Table 3b).

### 3.3. Secondary Outcomes

Comparison of lipid-related and lipid-independent risk factors for cardiovascular diseases in subjects with sufficient and insufficient vitamin C status

Among lipid-related markers, adults with insufficient plasma vitamin C had significantly higher proportion of elevated triglyceride (>150 mg/dL) and low HDL-C (<46 mg/dL). However, there was no difference in total cholesterol and LDL-C between groups. As for lipid-independent markers, there were greater proportions of adults with insufficient plasma vitamin C to have high homocysteine (>10 umol/L) and elevated hs-CRP (≥1 mg/dL) (both *p* < 0.001). However, no difference in lipoprotein(a) was noted between groups. (Table 4)

## 4. Discussion

Plasma vitamin C concentrations essentially depend on dietary vitamin C intake, intestinal absorption and renal excretion as well as diseases [27]. The strength of the current study was to provide plasma vitamin C information in adults receiving health examinations and not having diabetes/prediabetes and other chronic diseases (i.e., HIV infection, solid organ transplantation, chronic liver diseases and/or chronic renal failure). We discovered a high prevalence (39%) of insufficient plasma vitamin C in Taiwanese adults, despite the fact that Taiwan is a fruit kingdom [21] and the gross domestic product per capita in Taiwan was USD 22,000–25,000 in the study period (2018–2019). Our results confirm the literature findings that insufficient plasma vitamin C levels remain a current public health issue in many countries including Russia, America, New Zealand and Japan [35,44,45,46]. The cause for the high prevalence of vitamin C insufficiency may be due to unmet recommended dietary intakes in Taiwanese adults. A report based on 24 h dietary recall data from the Nutrition and Health Survey in Taiwan 2005–8 [47] showed that only 20% participants had the recommended number of servings for fruit and approximately 30% met that for vegetables from the Taiwan Food-Guide recommendations for adults [47]. Supplementation may be suggested for patients with insufficient plasma vitamin C.

Multivariate logistic regression analysis identified male gender, high BMI, age 20–39, and winter/spring as independent predictors of insufficient plasma vitamin C for all subjects in subtropical Taiwan. Epidemiological studies have frequently shown higher vitamin C concentrations in females than in males [48]. It may be attributed to more intakes of fruits and vegetables by females [49] as well as partly caused by a volumetric dilution effect due to differences in fat-free mass [50]. Among the health examiners, the young group (20–39 years of age) had lower levels of plasma vitamin C than the older group in this study. This was similar to the result of 2003–2004 National Health and Nutrition Examination Survey in the United States [48] showing that the young group (20–39 years of age) had the lower levels of vitamin C among supplement users [48]. It is likely that older subjects (>60 years of age) paid more attention to their health condition and used the supplements. Although gender and age are nonmodifiable factors, our findings suggest a need for public health interventions to decrease the prevalence of plasma vitamin C insufficiency in the male and young Taiwanese populations and to potentially decrease the risk of long-term adverse health effects.

Major food sources of vitamin C are fresh fruits and vegetables, in particular fresh fruits. Taiwan, located in a subtropical region, is a fruit kingdom, due to the suitable weather that is allows many kinds of fruits to be harvested during all four seasons [21]. In the present study, the highest prevalence (43.8%) of insufficient plasma vitamin C was observed in spring, followed by winter (41.3%), summer (37.3%) and autumn (34.7%). The results seemed to be associated with vitamin C amounts per 100 mg in seasonal fruits. The plasma vitamin C status may vary as a reflection of seasonal change in consumption of overall and particular fruits which depends on the availability and pricing [51]. It is rational that winter/spring independently predicted plasma vitamin C insufficiency due to lower vitamin C amounts in seasonal fruits. Given the higher risk of plasma vitamin C insufficiency in winter/spring, higher dietary vitamin C intakes or vitamin C supplements are required to ensure a sufficient vitamin C supply.

As for the implication of adiposity, high BMI (obesity and overweight) was identified as an independent predictor of insufficient plasma vitamin C among all subjects. Subgroup analysis by gender showed that both obesity and overweight females exhibited greater risk of having insufficient plasma vitamin C compared to females with normal BMI. In males, only obesity (BMI ≥ 27.5) not overweight (BMI 23–27.4) independently predicted for insufficient plasma vitamin C. The findings support previous results that plasma vitamin C was inversely related to BMI [50]. Effects of adiposity on plasma vitamin C were weaker in males due their lower percentage of body fat. Taken together, low consumption of vegetables and fruit as well as obesity were modifiable factors which should be intervened in to receive the benefits of sufficient plasma vitamin C.

In the current study, subjects with insufficient vitamin C status were found to have higher proportions of high triglyceride and low HDL-C significantly when compared to subjects with sufficient plasma vitamin C. However, there was no difference in total cholesterol and LDL-C between groups. Inconsistent with our findings, a previous study demonstrated that plasma levels of ascorbic acid in healthy elderly adults were not correlated with triglycerides, total cholesterol, LDL-C, and HDL-C [52]. However, a meta-analysis of randomized controlled trials shows that vitamin C supplementation with 500 mg daily for a minimum of 4 weeks significantly decreased plasma levels of triglycerides and LDL-C, but had no effective increase on HDL-C [53]. Results are inconsistent among previous studies and ours in examining plasma vitamin C concentrations and levels of dyslipidemia. Obviously, more studies are needed to elucidate the associations between levels of plasma vitamin C and dyslipidemia.

Hyperhomocysteinemia and elevated hs-CRP are well-known lipid-independent risk factors of cardiovascular diseases through increased oxidative stress [12,15,16] which can be reduced effectively by antioxidant effects of vitamin C [13]. In this study, adults with insufficient plasma vitamin C were found to have higher incidence of homocysteine > 10 umol/L and hs-CRP ≥ 1 mg/dL. Results not only support negative correlations between plasma vitamin C and levels of homocysteine/hs-CRP in clinical studies [17,54], but also provided the biomedical evidence for a long-term prospective cohort study showing a negative relationship between plasma vitamin C levels and heart disease mortality [55]. Although lipoprotein(a) was also a lipid-independent risk factor of cardiovascular diseases [3], we found no association between lipoprotein(a) and plasma vitamin C in contrast to a previous study discovering a negative correlation between lipoprotein(a) deposits in brain vessels and brain levels of vitamin C [56]. However, an intervention study revealed no significant lowering effect of vitamin C supplementation 4.5 g/day for 12 weeks on lipoprotein(a) levels in patients with coronary diseases [57]. Accordingly, more studies are needed to investigate the adverse effects of insufficient plasma vitamin C on these lipid-independent risk factors for cardiovascular diseases.

There were some limitations in the current study. First, we did not include data on vitamin C supplement and dietary intake from Food Frequency Questionnaire due to original data lacking this information. However, plasma vitamin C is a more precise index than data on vitamin C supplement and dietary intake for assessing the vitamin C status in human body [58]. Second, despite risk factors of vitamin C deficiency including smoking/alcohol use, we did not evaluate associations between smoking/alcohol use and vitamin C status [50]. Subjects undergoing health examinations were asked to complete a self-reported questionnaire concerning smoking and alcohol use. However, questionnaire completion was voluntary and many subjects chose not to answer these questions. Third, vitamin C status varies by race. Only Taiwanese were included in the study population. Further studies including various races are needed to confirm the findings.

## 5. Conclusions

A high prevalence of insufficient plasma vitamin C concentrations was found in adult subjects living in a subtropical region. Male gender, high BMI, age 20–39 and winter/spring independently predicted insufficient vitamin C for all subjects. The findings suggest there is a need for public health interventions to decrease the prevalence of plasma vitamin C insufficiency in the high-risk subjects. Future research has to be concerned about public health action plans for potentially reducing the higher rate of insufficient plasma vitamin C concentrations in winter/spring. Significantly greater proportions of subjects with insufficient plasma vitamin C had lower HDL-C levels and high levels of triglyceride, homocysteine and hs-CRP. However, there were no differences in total cholesterol, LDC-C and lipoprotein(a) levels between groups. Future studies are warranted to confirm the findings and to determine the underlying mechanisms of the causal links between plasma vitamin C status and these risk factors of cardiovascular diseases.

## Figures and Tables

**Table 1:** Demographic and anthropometric characteristics of the study population in a subtropical region.

		Vitamin C Status		
	Total (*n* = 3899) Mean (SD)	Sufficient (*n* = 2379, 61%) (≥50 µmol/L, ≥8.9 mg/L)	Insufficient (*n* = 1520, 39%) (<50 µmol/L, ≤8.8 mg/L)	*p*
Age, mean (SD), years	48.59 (11.92)	49.2 (11.9)	47.6 (11.8)	<0.001
BMI, mean (SD)	24.27 (3.48)	23.54 (3.54)	24.90 (3.86)	<0.001
Gender, mean (SD), mg/L		*n* (%)	*n* (%)	<0.001
Male	9.04 (3.09)	1143 (48.0%)	1044 (68.7%)	
Female	10.78 (3.14)	1236 (52.0%)	476 (31.3%)	
Vitamin C status in age groups, mean (SD), mg/L		*n* (%)	*n* (%)	0.001
20–39	9.47 (3.35)	531 (22.3%)	419 (27.6%)	
40–59	9.80 (3.16)	1357 (57.0%)	831 (54.7%)	
≥60	10.2 (3.26)	491 (20.7%)	270 (17.7%)	
Vitamin C status in seasons, mean (SD), mg/L (* Seasonal fruits and vitamin C amount per 100 g)		*n* (%)	*n* (%)	<0.001
Winter (* oranges 41.2 mg; apples 2.9 mg)	9.44 (3.07)	679 (58.7%)	478 (41.3%)	
Spring (* pineapples 12.0 mg; bananas 10.7 mg)	9.48 (2.97)	428 (56.2%)	333 (43.8%)	
Summer (* lychees 52.3 mg; mangoes 22.7 mg; pears 4.6 mg; grapes 3.8 mg)	10.04 (3.28)	506 (62.7%)	301 (37.3%)	
Autumn (* guavas 137.9 mg; papayas 58.3 mg; pomelos 54.5 mg; pitayas 5.3–6.3 mg)	10.19 (3.42)	766 (65.3%)	408 (34.7%)	
Vitamin C status in BMI groups, mean (SD), mg/L				<0.001
Obesity (≥27.5)	8.67 (3.2)	11.41 (2.19)	6.42 (2.00)	
Overweight (23–27.4)	9.56 (3.07)	11.55 (2.11)	6.73 (1.71)	
Normal (<23)	10.5 (3.23)	12.07 (2.28)	6.86 (1.87)	

SD—standard deviation; BMI—body mass index; *n*—number. Student *t*-test was used for the differences in continuous data between groups; chi-square test for the differences in categorical variables among groups. * Seasonal fruits and vitamin C amount per 100 g.

**Table 2:** Predictors for insufficient vitamin C levels (<50 µmol/L, ≤8.8 mg/L) among all subjects.

Vitamin C Status	Sufficient, *n* (%)	Insufficient, *n* (%)	Crude OR (95% CI)	*p*	AOR (95% CI)	*p*
All	2379 (61.0)	1520 (39.0)				
Gender						
Male	1143 (52.3)	1044 (47.7)	2.37 (2.07–2.71)	<0.001	2.14 (1.85–2.48)	<0.001
Female	1236 (72.2)	476 (27.8)	1.0		1.0	
Age groups						
20–39	531 (55.9)	419 (44.1)	1.44 (1.18–1.75)	<0.001	1.63 (1.33–1.99)	<0.001
40–59	1357 (62.0)	831 (38.0)	1.11 (0.94–1.32)	0.220	1.11 (0.93–1.33)	0.237
≥60	491 (64.5)	270 (35.5)	1.0		1.0	
Season						
Winter/Spring	1107 (57.7)	811 (42.3)	1.31 (1.16–1.50)	<0.001	1.37 (1.20–1.57)	<0.001
Summer/Autumn	1272 (64.2)	709 (35.8)	1.0		1.0	
BMI groups						
Obesity (≥27.5)	289 (45.2)	351 (54.8)	2.78 (2.30–3.35)	<0.001	2.13 (1.74–2.60)	<0.001
Overweight (23–27.4)	961 (58.7)	675 (41.3)	1.61 (1.39–1.86)	<0.001	1.30 (1.12–1.52)	<0.001
Normal (<23)	1129 (69.6)	494 (30.4)	1.0		1.0	

OR—odds ratio; BMI—body mass index; AOR—adjusted odds ratio. AOR—Adjusted Gender, Age groups, Season and BMI groups. Only variables with *p* < 0.10 in univariate logistic analyses were entered into a multivariate logistic regression.

**Table 3:** (**a**) Predictors for insufficient vitamin C levels (<50 µmol/L, ≤8.8 mg/L) among female adults. (**b**) Predictors for insufficient vitamin C levels (<50 µmol/L, ≤8.8 mg/L) among male adults.

**(a)**						
**Vitamin C Status**	**Sufficient, *n* (%)**	**Insufficient, *n* (%)**	**Crude OR (95% CI)**	***p***	**AOR (95% CI)**	***p***
Female	1236 (72.2)	476 (27.8)				
Age group						
20–39	313 (63.2)	182 (37.8)	1.94 (1.41–2.67)	<0.001	2.21 (1.59–3.06)	<0.001
40–59	676 (75.4)	220 (24.6)	1.09 (0.80–1.47)	0.590	1.17 (0.86–1.59)	0.319
≥60	247 (76.9)	74 (23.1)	1.0		1.0	
Season						
Winter + Spring	605 (68.6)	276 (32.4)	1.44 (1.16–1.78)	<0.001	1.47 (1.18–1.82)	<0.001
Summer + Autumn	631 (75.9)	200 (24.1)	1.0		1.0	
BMI groups						
Obesity (≥27.5)	80 (53.6)	69 (46.4)	2.65 (1.87–3.77)	<0.001	2.96 (2.07–4.24)	0.005
Overweight (23–27.4)	347 (70.6)	144 (29.4)	1.28 (1.01–1.62)	<0.001	1.42 (1.11–1.81)	<0.001
Normal (<23)	809 (75.4)	263 (24.6)	1.0		1.0	
**(b)**						
**Vitamin C Status**	**Sufficient, *n* (%)**	**Insufficient, *n* (%)**	**Crude OR (95% CI)**	***p***	**AOR (95% CI)**	***p***
Male	1143 (52.3)	1044 (47.7)				
Age group						
20–39	218 (47.9)	237 (52.1)	1.35 (1.04–1.76)	0.024	1.31 (1.01–1.71)	0.048
40–59	681 (52.4)	611 (47.6)	1.12 (0.90–1.39)	0.319	1.10 (0.88–1.37)	0.411
≥60	244 (55.4)	196 (44.6)	1.0		1.0	
Season						
Winter + Spring	502 (48.4)	535 (51.6)	1.34 (1.13–1.59)	<0.001	1.33 (1.12–1.57)	0.001
Summer + Autumn	641 (55.7)	509 (44.3)	1.0		1.0	
BMI groups						
Obesity (≥27.5)	209 (42.5)	282 (57.5)	1.87 (1.46–2.39)	<0.001	1.85 (1.44–2.37)	<0.001
Overweight (23–27.4)	614 (53.6)	531 (46.4)	1.20 (0.98–1.47)	0.085	1.21 (0.98–1.48)	0.073
Normal (<23)	320 (58.0)	231 (42.0)	1.0		1.0	

OR—odds ratio; BMI—body mass index; AOR—adjusted OR. AOR—Adjusted Gender, Age groups, Season and BMI groups. Only variables with *p* < 0.10 in univariate logistic analyses were entered into a multivariate logistic regression.

**Table 4:** Lipid-related and lipid-independent risk factors for cardiovascular diseases in subjects with sufficient and insufficient vitamin C.

	Vitamin C Status		
Risk Factors for Cardiovascular Diseases	Sufficient (*n* = 2379, 61%) Mean (SD)	Insufficient (*n* = 1520, 39%) Mean (SD)	*p*
Lipid-related marker			
Triglycerides, *n* (%)			<0.001
≤150 mg/dL	1886 (79.3)	1075 (71.2)	
>150	493 (20.7)	435 (28.8)	
Total cholesterol, *n* (%)			0.613
≤200 mg/dL	1317 (55.4)	854 (56.2)	
>200	1062 (44.6)	666 (43.8)	
HDL-C, *n* (%)			<0.001
≥46 mg/dL	1493 (62.7)	717 (47.2)	
<46	886 (37.3)	803 (52.8)	
LDL-C, *n* (%)			0.646
<100 mg/dL	495 (20.8)	307 (20.2)	
≥100	1884 (79.2)	1213 (79.8)	
Lipid-independent marker			
Lipoprotein(a), *n* (%)			0.761
<30 mg/dL	1908 (80.2)	1213 (79.8)	
≥30	471 (19.8)	307 (20.2)	
Homocysteine, *n* (%)			<0.001
≤10 umol/L	1789 (75.2)	763 (50.2)	
>10	590 (24.8)	757 (49.8)	
hs-CRP, *n* (%)			<0.001
<1.0 mg/L	1387 (58.3)	555 (36.5)	
≥1.0	992 (41.7)	965 (63.5)	

HDL-C—high-density lipoprotein cholesterol; LDL-C—low-density lipoprotein cholesterol; hs-CRP—high-sensitivity C-reactive protein. Chi-square test for the differences in categorical variables between groups.

## Data Availability

Anonymized data not published within this article will be made available and shared by request from any qualified investigator.
